# ATP8B2-Mediated Asymmetric Distribution of Plasmalogens Regulates Plasmalogen Homeostasis and Plays a Role in Intracellular Signaling

**DOI:** 10.3389/fmolb.2022.915457

**Published:** 2022-06-27

**Authors:** Masanori Honsho, Shiro Mawatari, Yukio Fujiki

**Affiliations:** ^1^ Department of Neuroinflammation and Brain Fatigue Science, Graduate School of Medical Sciences, Kyushu University, Fukuoka, Japan; ^2^ Institute of Rheological Functions of Food-Kyushu University Collaboration Program, Kyushu University, Fukuoka, Japan; ^3^ Institute of Rheological Functions of Food, Fukuoka, Japan; ^4^ Graduate School of Science, University of Hyogo, Hyogo, Japan

**Keywords:** plasmalogen, sensing, asymmetric distribution, P4-ATPase, Akt, plasma membrane

## Abstract

Plasmalogens are a subclass of glycerophospholipid containing vinyl-ether bond at the *sn*-1 position of glycerol backbone. Ethanolamine-containing plasmalogens (plasmalogens) are major constituents of cellular membranes in mammalian cells and *de novo* synthesis of plasmalogens largely contributes to the homeostasis of plasmalogens. Plasmalogen biosynthesis is regulated by a feedback mechanism that senses the plasmalogen level in the inner leaflet of the plasma membrane and regulates the stability of fatty acyl-CoA reductase 1 (Far1), a rate-limiting enzyme for plasmalogen biosynthesis. However, the molecular mechanism underlying the localization of plasmalogens in cytoplasmic leaflet of plasma membrane remains unknown. To address this issue, we attempted to identify a potential transporter of plasmalogens from the outer to the inner leaflet of plasma membrane by focusing on phospholipid flippases, type-IV P-type adenosine triphosphatases (P4-ATPase), localized in the plasma membranes. We herein show that knockdown of *ATP8B2* belonging to the class-1 P4-ATPase enhances localization of plasmalogens but not phosphatidylethanolamine in the extracellular leaflet and impairs plasmalogen-dependent degradation of Far1. Furthermore, phosphorylation of protein kinase B (AKT) is downregulated by lowering the expression of *ATP8B2*, which leads to suppression of cell growth. Taken together, these results suggest that enrichment of plasmalogens in the cytoplasmic leaflet of plasma membranes is mediated by ATP8B2 and this asymmetric distribution of plasmalogens is required for sensing plasmalogens as well as phosphorylation of AKT.

## Introduction

Alkenyl-ether glycerophospholipids characterized by the presence of a vinyl ether linkage at the *sn*-1 position are named plasmalogens, in which the head group of plasmalogens is commonly occupied by ethanolamine in mammals (Nagan and Zoeller, 2001; Braverman and Moser, 2012). Physiological roles of plasmalogens in the differentiation of Schwann cells, myelination of oligodendrocytes, neuron excitability, and homeostasis of cholesterol and neurotransmitter have been explored from several *in vitro* and *in vivo* studies using plasmalogen-deficient cells and mice (da Silva et al., 2014; Dorninger et al., 2019; Honsho et al., 2019; Malheiro et al., 2019; da Silva et al., 2021). Defect of plasmalogen biosynthesis caused by the mutations in genes essential for plasmalogen biosynthesis leads to extremely reduced levels of plasmalogens and causes a human genetic disorder, rhizomelic chondrodysplasia punctata (RCDP) (Wanders et al., 1992; Wanders et al., 1994; Braverman et al., 1997; Motley et al., 1997; Purdue et al., 1997; Buchert et al., 2014; Barøy et al., 2015). The patients with RCDP manifest severe growth retardation, proximal shortening of the upper extremities, and congenital cataract (Braverman and Moser, 2012). Plasmalogen levels of peripheral tissues in the RCDP model mouse are restored by the administration of alkylglycerol, by which the progression of the pathology in peripheral tissues such as testis and adipose tissue are suppressed (Brites et al., 2011), hence suggesting a physiological importance of the plasmalogen homeostasis in the functions of animal tissues.

The finding of a severe defect of plasmalogens in several different tissues from a patient with RCDP highlights that *de novo* synthesis of plasmalogens plays a pivotal role in the homeostasis of plasmalogens in tissues (Malheiro et al., 2015). Furthermore, recent findings of the elevation of plasmalogen biosynthesis in the patient caused by the mutation of fatty acyl-CoA reductase 1 (Far1), a rate-limiting enzyme of plasmalogen biosynthesis (Honsho et al., 2010) and sharing neurological symptoms such as spastic features, seizures with the patients defective in plasmalogen synthesis further strengthen the physiological importance of the regulation of plasmalogen biosynthesis (Ferdinandusse et al., 2021). These studies indicate that regulation of plasmalogen biosynthesis greatly contributes to the homeostasis of plasmalogens in tissues.

Plasmalogen biosynthesis is initiated in peroxisome matrix by acylation of dihydroxyacetonephosphate (DHAP) catalyzed by glyceronephosphate O-acyltransferase (GNPAT). The acyl-DHAP is further converted to alkyl-DHAP by alkylglycerone phosphate synthase (AGPS) that replaces acyl-chain of acyl-DHAP with long chain fatty alcohol (Nagan and Zoeller, 2001; Braverman and Moser, 2012). Far1, a peroxisomal C-tail anchored protein catalyzes the generation of fatty alcohol by reducing fatty acyl-CoA (Cheng and Russell, 2004). Based on the findings that Far1 activity is upregulated in the absence of plasmalogens (Honsho et al., 2010; Wiese et al., 2012), whereas plasmalogen synthesis is downregulated by the elevation of cellular plasmalogens by suppressing the activity of Far1 through augmenting degradation of Far1 without altering a transcription level of *FAR1* (Honsho et al., 2010), we proposed that Far1 is a rate-limiting enzyme in the plasmalogen biosynthesis. Given these results together with the recent study by Ferdinandusse et al. (2021), it is now widely accepted that biosynthesis of plasmalogens is regulated by modulating the stability of Far1 *via* sensing cellular plasmalogen level (Honsho and Fujiki, 2017). However, precise mechanism of sensing the plasmalogen level remains largely unknown.

To address this issue, we focused on the plasmalogens asymmetrically enriched in the inner leaflet of plasma membranes (Kirschner and Ganser, 1982; Fellmann et al., 1993; Honsho et al., 2017). This asymmetric distribution of plasmalogens is likely established by the function of type-IV P4-ATPase(s) that drives the outer-to-inner translocation of phospholipids. As reported (Honsho et al., 2017), the localization of plasmalogens in the outer leaflet of plasma membrane is indeed augmented upon reducing the protein level of CDC50A, a β-subunit of type-IV P4-ATPases that is essential for localization of the most of P4-ATPases in plasma membrane. Interestingly, the impairment of asymmetric distribution of plasmalogens elevates Far1 protein level (Honsho et al., 2017), thereby suggesting that asymmetric distribution of plasmalogens contributes to the regulation of plasmalogen biosynthesis. In addition to these findings, the recruitment of protein kinase B (AKT), a master regulator of cellular metabolism, to the plasma membranes that is an essential step for the phosphorylation of AKT is impaired in the absence of plasmalogens in Schwann cells, mouse embryonic fibroblasts, and neurons (da Silva et al., 2014; da Silva et al., 2021). This suggests that the asymmetric distribution of plasmalogens is tightly linked to cellular metabolism.

In the present study, we attempted to identify potential P4-ATPase(s) involved in translocating plasmalogens from extracellular leaflet to cytoplasmic leaflet and explore its functional significance in the biosynthesis and cellular functions of plasmalogens.

## Materials and Methods

### Cell Culture, DNA Transfection, and RNAi

HeLa cells were maintained in DMEM (Invitrogen) supplemented with 10% FBS (Sigma). All cell lines were cultured at 37°C under 5% CO_2_.

siRNA-mediated knockdown was performed using predesigned Stealth™ siRNAs (Invitrogen) and MISSION siRNAs (Sigma), respectively. Cells were harvested at 72 h after the initial transfection using Lipofectamine 2000. The following siRNAs were used: MISSION^®^ siRNA for human CDC50A (HS_TMEM30A_4522), ATP8B2#9 (SASI_Hs01_00037709), ATP8B2#13 (SASI_Hs01_00037913), ATP10D (SASI_Hs01_00069039), ATP11A (SASI_Hs01_00106358), ATP11B (SASI_Hs01_00045762), and Universal Negative Control#1. Stealth™ siRNAs for AGPS#30 (Agps-MSS213530) and AGPS#32 (AGPS-MSS213532). For counting cell numbers, cells were harvested at 48 and 72 h after transfection of siRNA and suspended in the equal volume of medium. An aliquot (30 μl) of the cell suspension was diluted with the equal volume of trypan blue (Fujifilm Wako Pure Chemical Corporation) and viable cells were counted with hemocytometer.

### Antibodies

We used rabbit antisera to AGPS (Honsho et al., 2008) and Far1 (Honsho et al., 2010). Rabbit antibodies to phospho-AKT (S473), phospho-AKT (T308), AKT were purchased from Cell Signaling. Rabbit antibody to ATP8B2 (Abgent) and mouse antibodies to actin (Santa Cruz) and GAPDH (Santa Cruz) were used.

### RT-PCR

Total RNA was isolated from HeLa cells using a TRIzol reagent (Ambion) and synthesis first-strand cDNA was performed using the PrimeScript RT reagent Kit (Takara Bio). Quantitative real-time RT-PCR was performed in an ABI7500 Real Time System (Applied Biosystem) using SYBR Premix Ex TaqTM II (Ti RNaseH Plus) (Takara Bio). Primers used were as follows: human ATP8B2 sense: ATP8B2Fw. 5′-TCC​TCC​TTT​CCA​GCA​GTG​AG-3′ antisense: ATP8B2Rv. 5′-ACT​TCA​CCG​TCA​AAC​TTG​GC-3′, human ATP10D sense: ATP10DFw. 5′-ACA​GTC​AGT​GGT​TCC​CTC​AG-3′ antisense: ATP10DRv. 5′-ACT​GGC​TCC​TTC​TCC​ACT​TC-3′, human ATP11A sense: ATP11AFw. 5′-GGC​TCC​GAA​CTT​TGT​GTG​TT-3′ antisense: ATP11ARv. 5′-TCT​CTC​GAT​CTT​GAA​GGG​CC-3′, and human ATP11B sense: ATP11BFw. 5′-GAC​TCC​ATG​TGC​TGT​TTC​CC-3′ antisense: ATP11BRv. 5′-ATC​TGC​TGA​TGT​GGG​TTG​GA-3’.

### Immunoblotting

Protein samples were separated by SDS-PAGE and electrotransferred to a nitrocellulose membrane (Bio-Rad). After blocking for 1 h in TBST (10 mM Tris-HCl pH 7.4, 200 mM NaCl, and 0.05% Tween-20) containing 5% non-fat dry milk, blots were subjected to immunoblotting with primary antibodies overnight at 4°C, followed by incubating with a secondary antibody for 2 h at room temperature. Immunoblots were developed with Clarity™ Western ECL Substrate (Bio-Rad) and scanned with an ImageQuant LAS 4010 imager (GE Healthcare). For antibody reprobing, membranes were incubated three times for 10 min in a mild membrane stripping buffer (Abcam), extensively washed with TBST, and incubated with antibody. The intensity of bands was quantified by ImageJ software (National Institutes of Health).

### Lipid Analysis

Distribution of plasmalogens in plasma membranes was assessed as described (Honsho et al., 2017). In brief, cells were metabolically labeled for 18 h with 0.1 µCi of ^14^C-ethanolamine (Moravek), washed with ice-cold SHT buffer (0.25 M sucrose, 10 mM Hepes-KOH, pH 8.5) containing 1 μg/ml taxol (Sigma), incubated on ice for 5  min, and further treated with 10 mM 2,4,6-trinitrobenzene sulfonic acid (TNBS) (Wako) dissolved in SHT buffer on ice for 30 min in the dark. Excess TNBS was quenched for 15 min with 50 mM Tris-HCl, pH 8.0. Equal aliquots (100 µg protein) of cell lysates were treated with 5% of trichloroacetic acid for 10 min at room temperature and precipitated by centrifugation at 20,000 × g for 1 min, followed by lipid extraction by the Bligh and Dyer method (Bligh and Dyer, 1959). Lipids were analyzed on thin layer chromatography plates (silica gel 60, Merck) with chloroform/methanol/acetic acid solution (v/v/v: 65/25/10) (Honsho et al., 2008). ^14^C-labeled lipids were detected by autoradiography using a FLA-5000 imaging analyzer (Fuji Film) and quantified using an image analyzer software (Multi Gauge, Fuji Film).

Cellular plasmalogen level was determined by liquid chromatography connected to tandem mass spectrometry (LC-MS/MS) with modification of the method described by Abe et al. (2014). Total cellular lipids were extracted by the Bligh and Dyer method (Bligh and Dyer, 1959). Briefly, cell lysates containing 50 µg of total cellular proteins were dissolved in methanol/chloroform/water (v/v/v: 2:1:0.8) and 50 pmol internal standard (1-heptadecanoyl-sn-glycero-3-phosphocholine, 1, 2-didodecanoyl-sn-glycero-3-phosphocholine and 1, 2-didodecanoyl-sn-glycero-3-phosphoethanolamine). After 5 min, 1 ml each of water and chloroform was added and the whole mixtures were centrifuged to collect the lower organic phase. One ml chloroform was added again to re-extract the lipids. Collected organic phase was then evaporated under the nitrogen stream and suspended in pure methanol. LC-MS assay was performed using a Xevo TQ-S micro with ACQUITY UPLC System (Waters). Samples were injected into an ACQUIRY UPLC BEH C18 column and then directly subjected to ESI-MS/MS analysis. A 10-μl aliquot of each sample was directly introduced by autosampler injector and the samples were separated by step-gradient elution with mobile phase A (acetonitrile:methanol:water at 2:2:1 (v/v/v), 0.1% formic acid and 0.028% ammonium) and mobile phase B (isopropanol, 0.1% formic acid and 0.028% ammonium) at the ratios: 100:0 (for 0–5 min), 70:30 (5–20 min), 45:55 (20–40 min), and 100:0 (40–51 min), with a flow rate (70 μl/min at 35°C) and source temperature (150°C). Ethanolamine plasmalogens (PlsEtns) were detected at positive ion mode and the method used to detect plasmalogens is listed in Supplementary Table S1. The data were analyzed and quantified using the TargetLynx (Waters).

### Statistical Analysis and Data Presentation

Statistical analysis was performed using one-tailed Student’s t-tests unless otherwise described in figure legends. A *p* < 0.05 was considered statistically significant. Quantitative data were shown as means ± SD.

## Results

### Identification of P4-ATPase Responsible for Topogenesis of Plasmalogens

We earlier showed that asymmetric distribution of plasmalogens in HeLa cells is compromised upon knocking down of CDC50A, a β-subunit of P4-ATPases (Takatsu et al., 2011), resulting in the elevation of Far1 protein level (Honsho et al., 2017). Given these results together with the fact that several P4-ATPases require the association with CDC50A protein for their exit from the endoplasmic reticulum (Bryde et al., 2010; Paulusma and Elferink, 2010; van der Velden et al., 2010), we hypothesized that asymmetric distribution of plasmalogens in plasma membrane is established by the function of P4-ATPase(s). Therefore, we attempted to identify the P4-ATPase(s) responsible for topogenesis of plasmalogens by focusing on P4-ATPases expressed in HeLa cells.

Of the eleven P4-ATPase that are expressed in HeLa cells (Takatsu et al., 2011), we selected four P4-ATPases (ATP8B2, ATP10D, ATP11A, and ATP11B) that associate with CDC50A (Shin and Takatsu, 2019) and present on plasma membranes (Andersen et al., 2016), whereas ATP8B1 and ATP11C were not considered here from potential P4-ATPases for plasmalogens because of their functions in transport of phosphatidylcholine (PC) and phosphatidylserine (PS), respectively (Takatsu et al., 2014; Takada et al., 2015; Hayashi et al., 2018).

**FIGURE 1:**
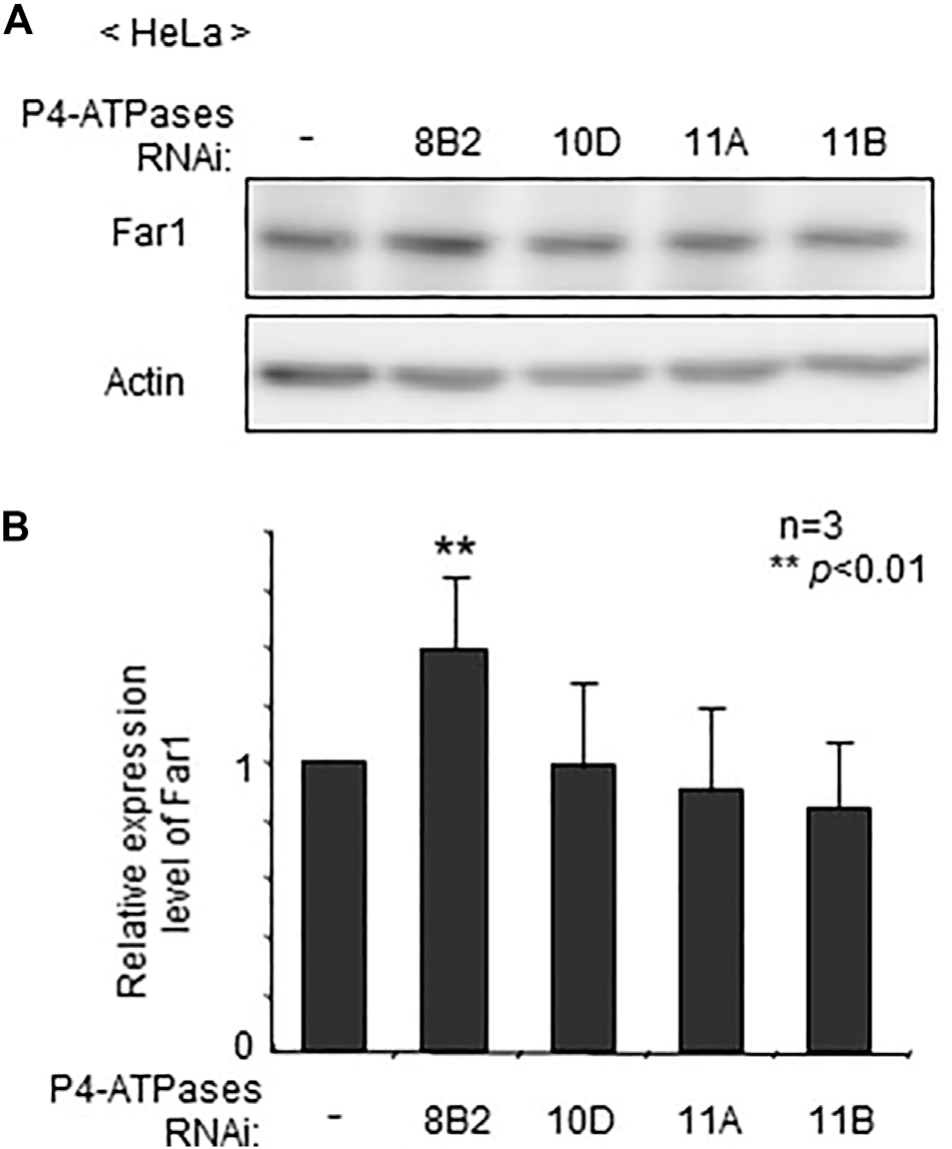
Elevated protein level of Far1 by knockdown of *ATP8B2*. **(A)** HeLa cells were transfected with dsRNA against *ATP8B2* (8B2), *ATP10D* (10D), *ATP11A* (11A), or *ATP11B* (11B) and cultured for 72 h. Protein level of Far1 and actin (Actin) was analyzed by immunoblotting with respective antibodies as indicated. Actin was used as a loading control. **(B)** The amount of Far1 was normalized by the actin level in respective transfectants and presented relative to that in non-transfected cells (-) (*n* = 3). Data indicate means ± SD. **p < 0.01 by Student’s *t*-test.

To assess whether localization of plasmalogens in the inner leaflet of plasma membranes is mediated by any of these four P4-ATPases, we verified protein level of Far1 in HeLa cells that had been treated for knocking down of P4-ATPases. The transfection of siRNAs against four P4-ATPases successfully reduced the expression of the target genes as assessed at mRNA level (Supplementary Figure S1). The elevation of Far1 protein levels was detected upon transfection of siRNA against *ATP8B2* but not other *P4-ATPases* (Figure 1). These results suggest that the plasmalogen-sensing mechanism regulating the levels of Far1 is affected by the downregulation of ATP8B2.

**FIGURE 2:**
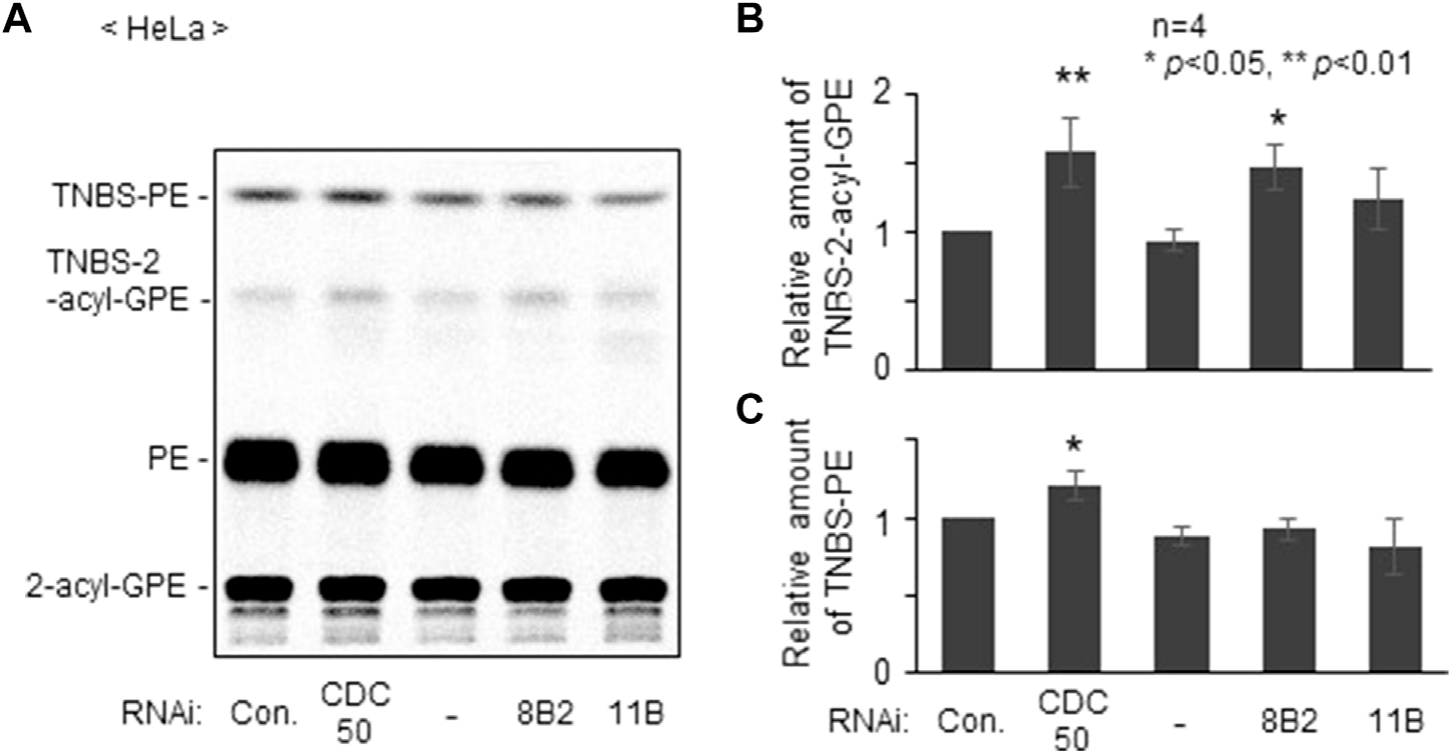
Knockdown of *ATP8B2* elevates plasmalogens localization in outer leaflet of plasma membranes. **(A)** HeLa cells were transfected for 54 h with control dsRNA (Con.) and dsRNA against *CDC50A* (CDC50), *ATP8B2* (8B2), or *ATP11B* (11B), and further cultured in the presence of ^14^C-ethanolamine for 18 h. Cells were incubated with TNBS, a membrane impermeable amine-reactive reagent. Total cellular lipids were extracted from equal aliquots of respective cell proteins after treatment of trichloroacetic acid to cleave vinyl-ether bond of plasmalogens and analyzed by TLC. Note that plasmalogens and TNBS-modified plasmalogens are converted to 2-acyl-GPE and TNBS-2-acyl-GPE, respectively, upon trichloroacetic acid treatment. **(B,C)** Relative amounts of TNBS-modified plasmalogens **(B)** and TNBS-modified PE **(C)** were represented by taking as one those in HeLa cells transfected with control dsRNA (Con.) (*n* = 4). Data indicate means ± SD. *p < 0.05, **p < 0.01, by one-way ANOVA with Dunnett’s post hoc test as compared with control dsRNA-transfected HeLa cells (Con.).

We next studied distribution of plasmalogens at the outer leaflet in *ATP8B2*-knocked down HeLa cells with a membrane impermeable amine-reactive reagent TNBS, by which phosphatidylethanolamine (PE) and plasmalogens localizing at the outer leaflet of plasma membranes are converted to TNBS-PE and TNBS-plasmalogens distinguishable from PE and plasmalogens (Kobayashi and Pagano, 1989; Honsho et al., 2017). By acid hydrolysis of vinyl-ether bond of plasmalogens, TNBS-plasmalogens and plasmalogens are converted to TNBS-2-acyl-glycerophosphoethanolamine (GPE) and 2-acyl-GPE, respectively, thereby allowing separation of PE, plasmalogens, and two TNBS-modified ethanolamine glycerophospholipids by thin layer chromatography (Honsho et al., 2017). As anticipated, the amount of TNBS-plasmalogens but not TNBS-PE was indeed elevated in *ATP8B2*-knocked down HeLa cells, whereas distribution of plasmalogens and PE was not altered in *ATP11B*-knocked down cells (Figure 2). Taken together, these results suggested that ATP8B2 flips plasmalogens from the exoplasmic to the cytosolic leaflet at the plasma membranes and a less mount of plasmalogens in the inner leaflet of plasma membrane induces the elevation of Far1 protein due to the impairment of sensing plasmalogens.

### Plasmalogens Are Required for the Phosphorylation of AKT

The physiological roles of plasmalogens localized in the inner leaflet of plasma membranes remain largely unknown. The fact that recruitment of AKT to the plasma membranes, a critical step in its phosphorylation by phosphoinositide-dependent protein kinase one and mammalian TOR complex 2 (Vivanco and Sawyers, 2002; Bozulic and Hemmings, 2009; Gao et al., 2011) is impaired in the absence of plasmalogens in mouse embryonic fibroblasts, Schwan cells, and neurons derived from *Gnpat* ^−/-^ mice (da Silva et al., 2014; da Silva et al., 2021), suggests the link of asymmetric distribution of plasmalogens and cellular metabolism.

**FIGURE 3:**
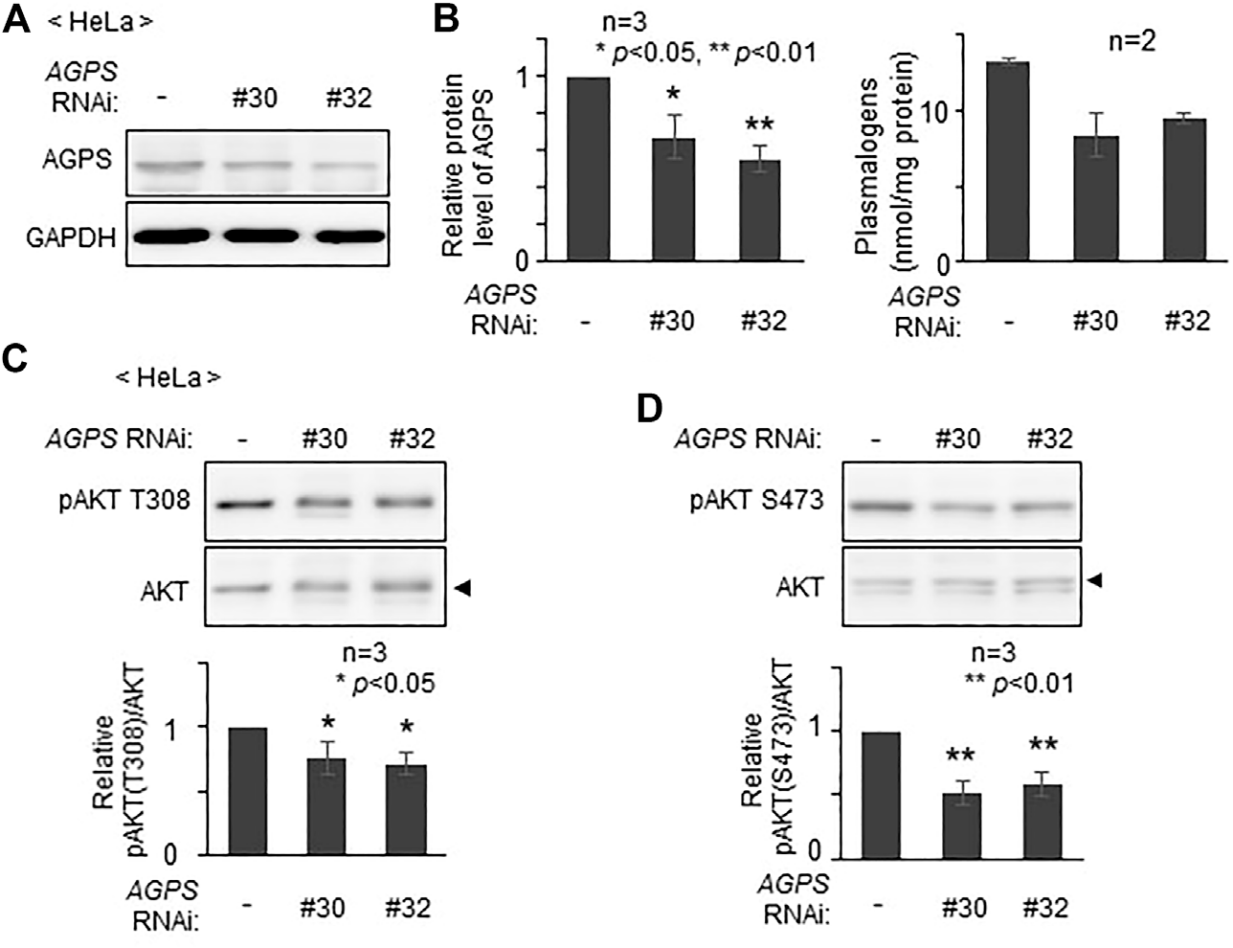
Reduction of AKT activity by lowering plasmalogens. **(A)** HeLa cells were transfected with two different dsRNAs (#30 and #32) against *AGPS* and cultured for 72 h. Protein level of AGPS was analyzed by immunoblotting. GAPDH was used as a loading control. **(B)** Protein level of AGPS (*left*) and plasmalogen level (*right*) were represented by taking as one that in non-transfected HeLa cells. **(C,D)** Phosphorylation of AKT was analyzed by immunoblotting using antibodies specific for the phosphorylated T308 **(C)** and S473 **(D)** and total unmodified AKT (arrowhead). The amounts of pAKT T308 **(C)** and pAKT S473 **(D)** relative to total AKT were represented (*lower panels*). Data indicate means ± SD (*n* = 3). *p < 0.05 and *p < 0.01, by one-way ANOVA with Dunnett’s post hoc test as compared with HeLa cells (-).

To assess whether plasmalogens are required for the phosphorylation of AKT in other types of cells, we verified phosphorylation state of AKT by lowering the level of plasmalogens in HeLa cells. Upon knocking down *AGPS* with two different double-strand RNAs, AGPS protein was lowered to about 60–70% of that in mock-treated cells (Figures 3A,B) and plasmalogen level was reduced by nearly 30% (Figure 3B). Under these conditions, phosphorylation of AKT at both threonine 308 (T308) in the kinase domain and at serine 473 (S473) in the C-terminal regulatory domain was suppressed (Figures 3C,D). These results are consistent with the previous study showing the impaired AKT-activation in plasmalogen-deficient cells and mouse (da Silva et al., 2014; da Silva et al., 2021) and further reveal the importance of plasmalogen homeostasis in the AKT activation.

### Phosphorylation of AKT is Suppressed by a Reduced Expression of ATP8B2

**FIGURE 4:**
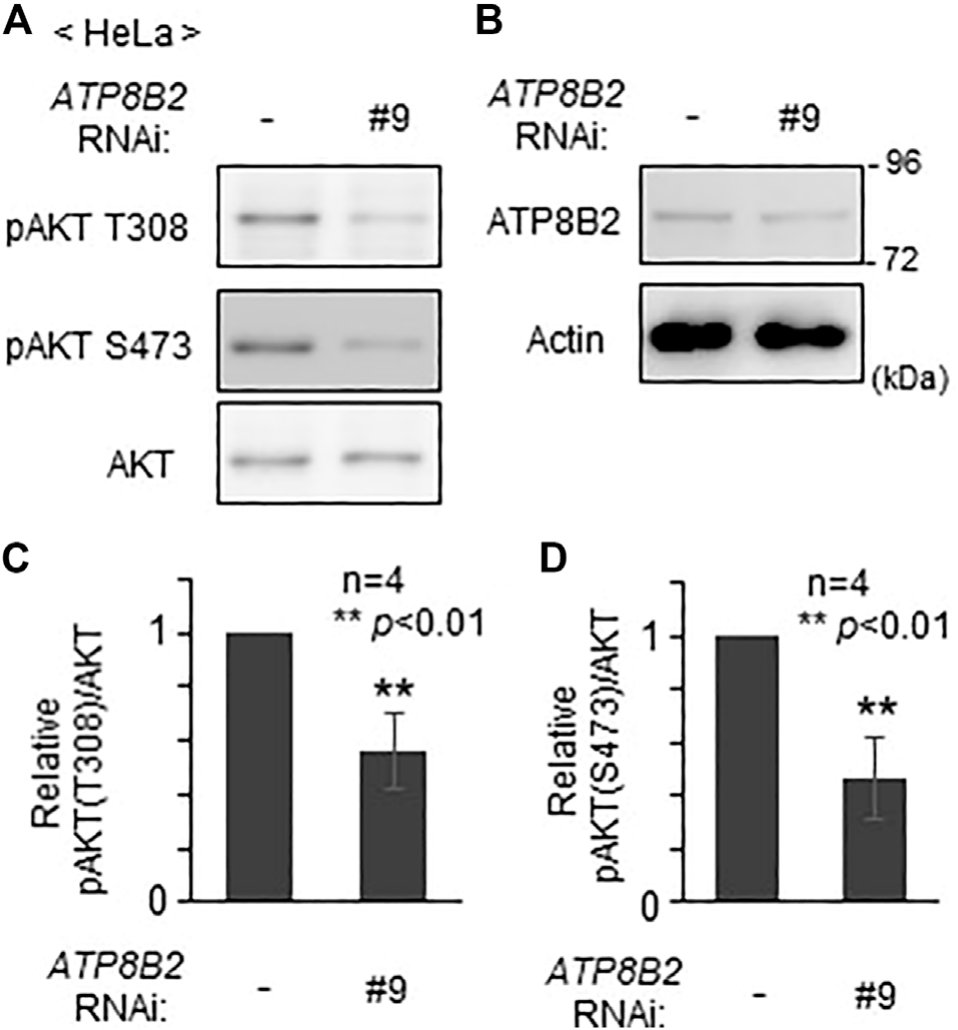
Reduction of AKT activity by lowering ATP8B2 expression. **(A,B)** HeLa cells were transfected with siRNA (#9) against *ATP8B2* and cultured for 72 h. Phosphorylation of AKT **(A)** and expression of ATP8B2 **(B)** were verified by immunoblot using the indicated antibodies. Actin was a loading control. **(C,D)** The amounts of pAKT T308 **(C)** and pAKT S473 **(D)** relative to total AKT were represented (*n* = 4). Data indicate means ± SD. **p < 0.01, by Student’s *t*-test.

We next investigated whether plasmalogens in the cytoplasmic leaflet of plasma membranes are required for the activation of AKT. To this end, we verified the effect of the knocking down of *ATP8B2* on the phosphorylation level of AKT. Phosphorylation of AKT at both threonine 308 and serine 473 was reduced upon transfection of siRNA#9 against *ATP8B2* (Figure 4), presumably giving rise to an impaired asymmetric distribution of plasmalogens (Figure 2). Likewise, phosphorylation of AKT was significantly less efficient in HeLa cells transfected with other *ATP8B2* siRNA#13 (Supplementary Figure S2), implying an impaired phosphorylation of AKT caused by mislocalization of plasmalogen from the inner leaflet of plasma membrane. Collectively, these results strongly suggest that ATP8B2-mediated localization of plasmalogens in the inner leaflet of plasma membrane linked to phosphorylation of AKT.

### Knockdown of ATP8B2 Suppresses Cell Growth

**FIGURE 5:**
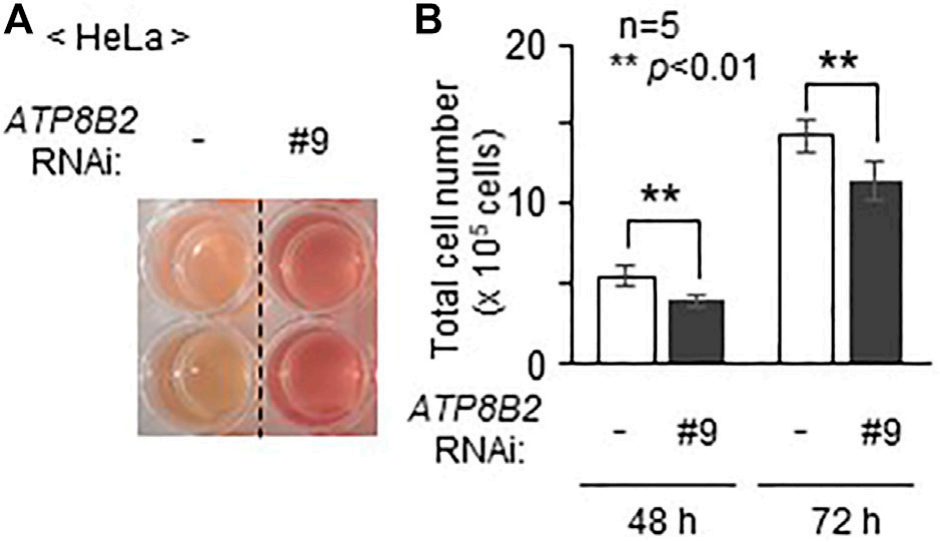
Suppression of cell growth upon lowering the expression of *ATP8B2*. **(A)** Mock transfected HeLa cells (-) or HeLa cells transfected with siRNA against *ATP8B2* (#9) were cultured for 72 h. Acidification of the culture medium was monitored by visual-checking the color of the medium. **(B)** Total number of cells cultured as in **(A)** was counted at the indicated time after transfection with the respective siRNA (*n* = 5). Data indicate means ± SD. **p < 0.01, by Student’s *t*-test.

Inhibition of AKT activity in several cancer cell lines induces cell death (Kostaras et al., 2020). In addition, suppression of AGPS activity in cancer cells such as MDA-MB-231 expressing a higher level of AGPS reduces cell proliferation, although the precise mechanism underlying the suppression of cell growth by the inhibition of AGPS activity remains unknown (Stazi et al., 2019). Moreover, we frequently observed less acidification of the culture medium in cells transfected with siRNA against *ATP8B2* expression as compared with control cells (Figure 5A), suggesting the reduced cell growth by lowering *ATP8B2* expression. Consistent with this result, total number of the *ATP8B2* siRNA-transfected cells was less than that in control siRNA-treated cells (Figure 5B). Taken together, these results suggest that the reduced expression of ATP8B2 lowers plasmalogens in the cytoplasmic leaflet of plasma membranes, thereby reducing the cell growth *via* suppressing AKT activation.

## Discussion

Asymmetric distribution of plasmalogens in the plasma membrane has been reported in red blood, myelin, and cultured cells (Kirschner and Ganser, 1982; Fellmann et al., 1993; Honsho et al., 2017). We earlier showed that asymmetric distribution of plasmalogens is important for the regulation of plasmalogen synthesis, where plasmalogens enriched in the inner leaflet of plasma membranes are sensed by yet unknown mechanism (Honsho et al., 2017). We here provide several lines of evidence that asymmetric distribution of plasmalogens is mediated by ATP8B2, a type-IV P4-ATPase (Figure 2). Furthermore, we demonstrated a role of plasmalogens enriched in the cytoplasmic leaflet of plasma membranes by showing the reduced activation of AKT in cells upon transfection of siRNA against *ATP8B2* (Figure 4; Supplementary Figure S2).

In the human protein atlas (https://www.proteinatlas.org/), ATP8B2 is described to be expressed in a wide range of tissues including brain, kidney, and lung where plasmalogens are enriched (Braverman and Moser, 2012). These data and the findings reporting an elevated protein level of Far1 in the kidney of plasmalogen-deficient *PEX7* knockout mouse (Wiese et al., 2012) and the cerebellum of plasmalogen-deficient *GNPAT* knockout mouse (Honsho et al., 2019) suggest that ATP8B2 is involved in the regulation of plasmalogen synthesis in these tissues.

ATP8B2 is reported to have flippase activity toward PC with a substrate, a fluorescent PC analogue, 1-oleoyl-2-{6-[(7-nitro-2-,3-benzoxadiazol-4-yl)amino]hexanoyl}-*sn*-glycero-3-phosphocholine (Takatsu et al., 2014), although its PC flippase activity is lower than that of ATP8B1 and ATP10A (Takatsu et al., 2014; Naito et al., 2015). Substrate specificity of ATP8B2 was assessed with several fluorescent phospholipids (Takatsu et al., 2014). However, no fluorescent-conjugated plasmalogen is commercially available, thus the topogenesis of plasmalogens has not been addressed despite being one of the major glycerophospholipids in mammalian cells. We here show that ATP8B2 functions as a flippase of plasmalogens by assessing the topology of chemically modified plasmalogens in the extracellular leaflet of plasma membranes (Figure 2). Clearly, developing a plasmalogen analogue such as fluorescent-conjugated plasmalogen is required for further investigation of the mechanistic insight to ATP8B2-mediated topogenesis of plasmalogens and identification of other P4-ATPases acting as another plasmalogen flippase, if any.

Physiological importance of plasmalogens enriched in the cytoplasmic leaflet of plasma membranes is not fully understood. We earlier proposed that topogenesis of plasmalogens is important for sensing plasmalogens from the finding that knockdown of *CDC50A* elevates the protein level of Far1 by compromising asymmetric distribution of plasmalogens (Honsho et al., 2017). This notion is further supported in the present study by the finding that a reduced expression of ATP8B2 induces the elevation of plasmalogens localized at the extracellular leaflet of plasma membranes, thereby increasing the Far1 protein level (Figures 1, 2).

Synthesis of plasmalogen can be augmented by expression of the mutant Far1 harboring a mutation in the regulatory region that is essential for the plasmalogen-induced degradation (Ferdinandusse et al., 2021) or by ectopic expression of wild-type Far1 but not GNPAT (Hajra et al., 2000; Honsho et al., 2010; Liu et al., 2005). Elevation of plasmalogens has been shown in several cancer cells (Albert and Anderson, 1977; Roos and Choppin, 1984; Snyder and Wood, 1969), which is likely associated with the pathogenicity of tumors because an inhibitor of AGPS lowers the plasmalogen levels and suppresses the pathogenicity of various types of cancer cells *in vitro* (Piano et al., 2015). According to the data from GEPIA 2 (http://gepia2.cancer-pku.cn/), expression of *TMEM189* encoding plasmanylethanolamine desaturase that introduces the vinyl ether double-bond into plasmanylethanolamine (Gallego-García et al., 2019; Werner et al., 2020) is upregulated in most of the human cancers, although higher expression of TMEM189 reduces the protein level of Far1 (Cui et al., 2021). In contrast to the upregulated expression of *TMEM189*, *ATP8B2* expression is reduced in most of those human cancers as searched in the GEPIA 2. Based on these facts, it is tempting to speculate that elevation of plasmalogens in human cancers is achieved by escaping from the plasmalogen-sensing step *via* reducing the plasmalogens in the cytoplasmic leaflet of plasma membrane. The higher level of plasmalogens augments the sensitivity of cancer cells to ferroptosis (Cui et al., 2021). Nevertheless, several different cancer cells get sensitized to evasion from ferroptosis by lowering plasmalogens where expression of *ATP8B2* mRNA is increased, though both protein and mRNA expressions of AGPS and TMEM189 are reduced (Zou et al., 2020). These results suggest that ATP8B2 is involved in the downregulation of plasmalogens by lowering the protein level of Far1 under pathophysiological condition together with the reduction in the expression of enzymes catalyzing plasmalogen biosynthesis.

AKT activation is reduced by lowering expression of ATP8B2 (Figure 4; Supplementary Figure S2), consistent with the earlier report indicating the impaired AKT activation in plasmalogen-deficient mouse embryonic fibroblasts, Schwann cells, and neurons (da Silva et al., 2014; da Silva et al., 2021). In our assay, PE was not exposed to the extracellular leaflet in *ATP8B2*-knocked down cells (Figure 2), which may be due to the compensatory function of other P4-ATPases such as ATP11A and ATP11C, both with a higher PE flipping activity than ATP8B2 (Segawa et al., 2016). In contrast, the exposure of PE in the extracellular leaflet was evident upon knocking down of *CDC50A* expression consistent with the earlier studies (Honsho et al., 2017; Kato et al., 2013) (Figure 2), which is likely due to the inactivation of several P4-ATPases simultaneously acting as PE flippase. Similarly, three P4-ATPases, ATP8B1, ATP10A, and ATP8B2, are a flippase towards PC in HeLa cells (Takatsu et al., 2011; Takatsu et al., 2014; Naito et al., 2015), of which the former two are suspected to compensate the PC flippase activity in cells reduced in the expression of ATP8B2. In a very recent study, PS is revealed as the most favored substrate for ATP8B1 (Cheng et al., 2022). Based on these facts together with a higher similarity of amino acid sequence between ATP8B2 and ATP8B1, ATP8B2 has a potential activity to transfer PS across membrane. The reduced activity of ATP8B2 toward PS might suppress AKT phosphorylation because PS is known to activate AKT (Huang et al., 2011). However, PS exposure in extracellular leaflet is unlikely in *ATP8B2*-knocked down cells, because plasma membrane-localized ATP11A and ATP11C, both have much higher PS-flipping activity as compared with ATP8B2 (Segawa et al., 2016), are expressed in HeLa cells as well as several human and mouse tissues (Takatsu et al., 2011). Taken together, these findings strongly suggest that the lower activation of AKT in *ATP8B2*-knocked down HeLa cells is most likely caused by the impaired asymmetric distribution of plasmalogens, hence strengthening the physiological consequence of asymmetrically distributed plasmalogens in cellular metabolism.

The possible link between ATP8B2 and Parkinson’s disease (PD) progression has been addressed by a genome-wide association study on PD progression using more than three thousand patients with PD (Tan et al., 2021). Noteworthily, the abrogated AKT activation is detectable in postmortem tissue from the substantia nigra of PD patients (Malagelada et al., 2008; Timmons et al., 2009), whereas the expression of constitutive active form of AKT blocks apoptosis of dopamine neurons of substantia nigra in a neurotoxin mouse model of Parkinsonism upon injecting 6-hydroxydopamine, an endogenous metabolite of dopamine (Ries et al., 2006). Based on these studies, we hypothesize that impairment of asymmetric distribution of plasmalogens mediated by ATP8B2 in dopamine neuron is a potential cause of the PD pathogenesis. Nevertheless, further investigations to address plasmalogen homeostasis, including the activity and expression levels of proteins involved in the regulation of plasmalogen biosynthesis, are required to understand how abnormalities in plasmalogen homeostasis are responsible for the pathogenesis of PD.

## Data Availability

The original contributions presented in the study are included in the article/Supplementary Material, further inquiries can be directed to the corresponding authors.
