# Supplementary material for: ATP8B2-Mediated Asymmetric Distribution of Plasmalogens Regulates Plasmalogen Homeostasis and Plays a Role in Intracellular Signaling
Source: Front Mol Biosci. 2022 Jun 27;9:915457. doi: 10.3389/fmolb.2022.915457 (PMC9271795; doi:10.3389/fmolb.2022.915457)

## Slide 1
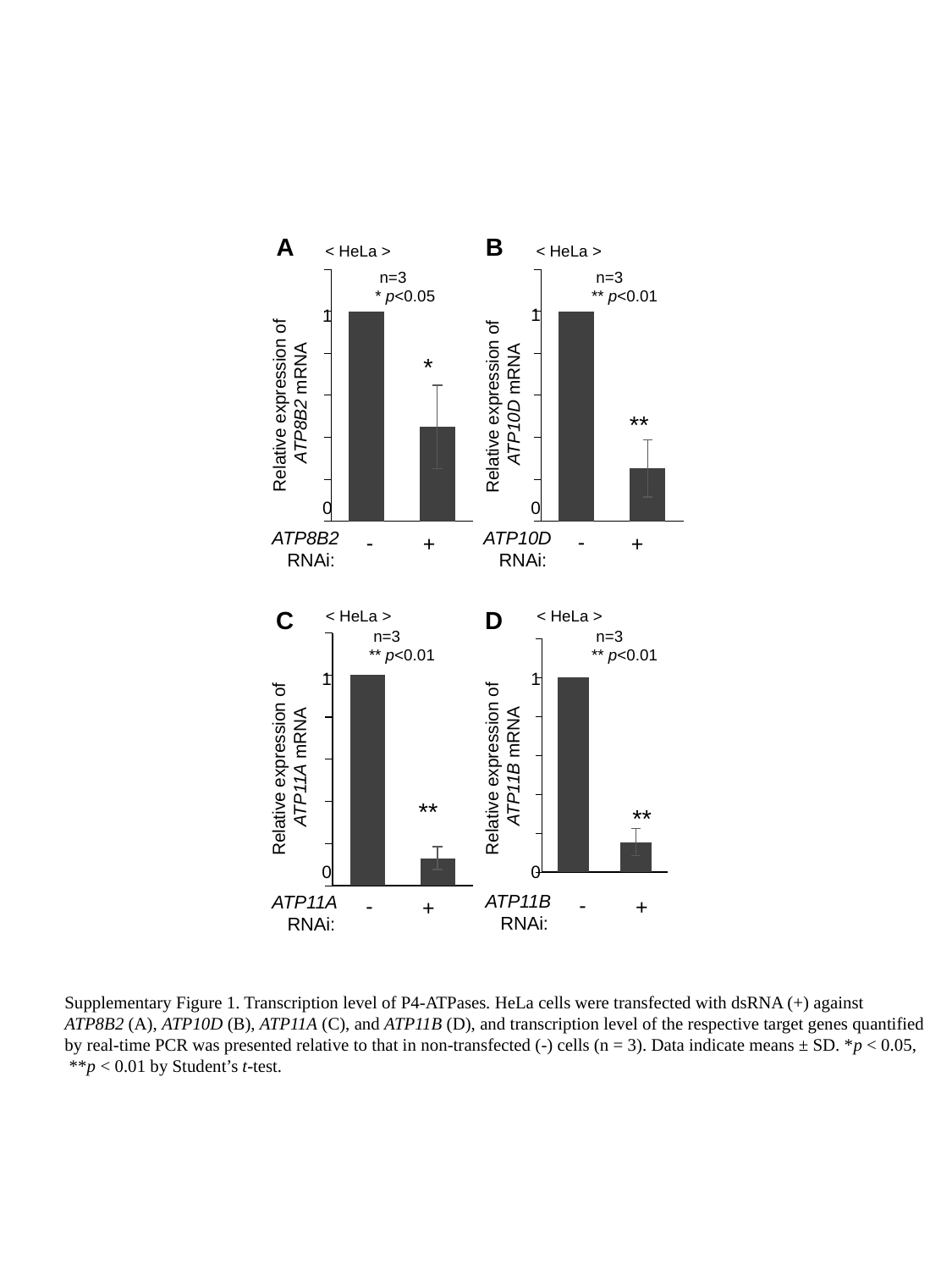

A
B
< HeLa >
< HeLa >
### Chart
| Category | |
|---|---|
### Chart
| Category | |
|---|---| n=3
* p<0.05
 n=3
** p<0.01
1
1
*
Relative expression of
ATP8B2 mRNA
Relative expression of
ATP10D mRNA
**
0
0
ATP10D
 RNAi:
ATP8B2
 RNAi:
-
-
+
+
C
D
< HeLa >
< HeLa >
### Chart
| Category | |
|---|---| n=3
** p<0.01
 n=3
** p<0.01
### Chart
| Category | |
|---|---|1
1
Relative expression of
ATP11B mRNA
Relative expression of
ATP11A mRNA
**
**
0
0
ATP11B
 RNAi:
ATP11A
 RNAi:
-
-
+
+
Supplementary Figure 1. Transcription level of P4-ATPases. HeLa cells were transfected with dsRNA (+) against
ATP8B2 (A), ATP10D (B), ATP11A (C), and ATP11B (D), and transcription level of the respective target genes quantified
by real-time PCR was presented relative to that in non-transfected (-) cells (n = 3). Data indicate means ± SD. *p < 0.05,
 **p < 0.01 by Student’s t-test.

## Slide 2
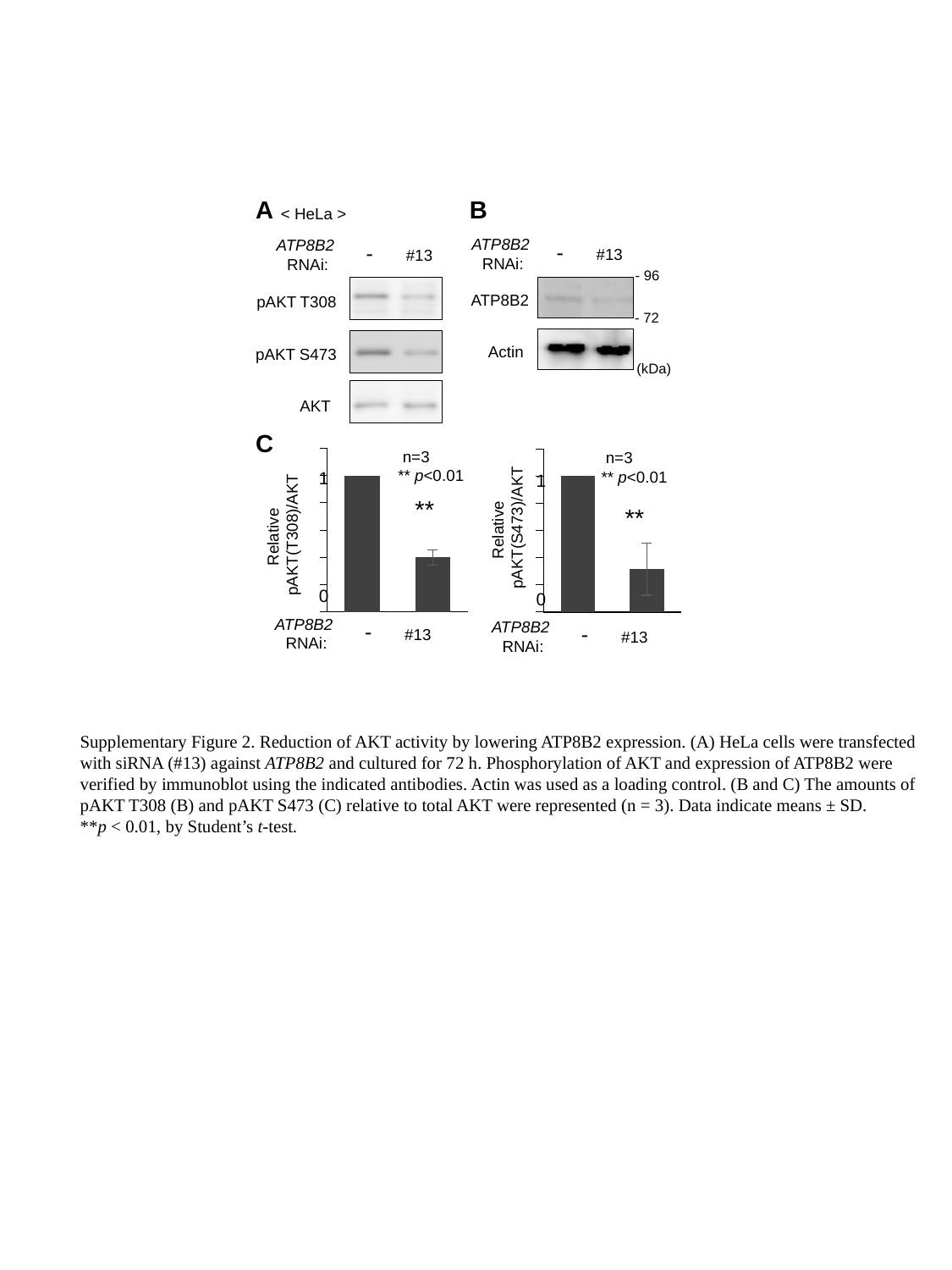

A
B
< HeLa >
ATP8B2
RNAi:
ATP8B2
RNAi:
-
-
#13
#13
- 96
ATP8B2
pAKT T308
- 72
Actin
pAKT S473
(kDa)
AKT
C
### Chart
| Category | |
|---|---|
### Chart
| Category | |
|---|---| n=3
** p<0.01
 n=3
** p<0.01
1
1
**
**
Relative
 pAKT(S473)/AKT
Relative
pAKT(T308)/AKT
0
0
ATP8B2
RNAi:
ATP8B2
RNAi:
-
-
#13
#13
Supplementary Figure 2. Reduction of AKT activity by lowering ATP8B2 expression. (A) HeLa cells were transfected
with siRNA (#13) against ATP8B2 and cultured for 72 h. Phosphorylation of AKT and expression of ATP8B2 were
verified by immunoblot using the indicated antibodies. Actin was used as a loading control. (B and C) The amounts of
pAKT T308 (B) and pAKT S473 (C) relative to total AKT were represented (n = 3). Data indicate means ± SD.
**p < 0.01, by Student’s t-test.

## Slide 3
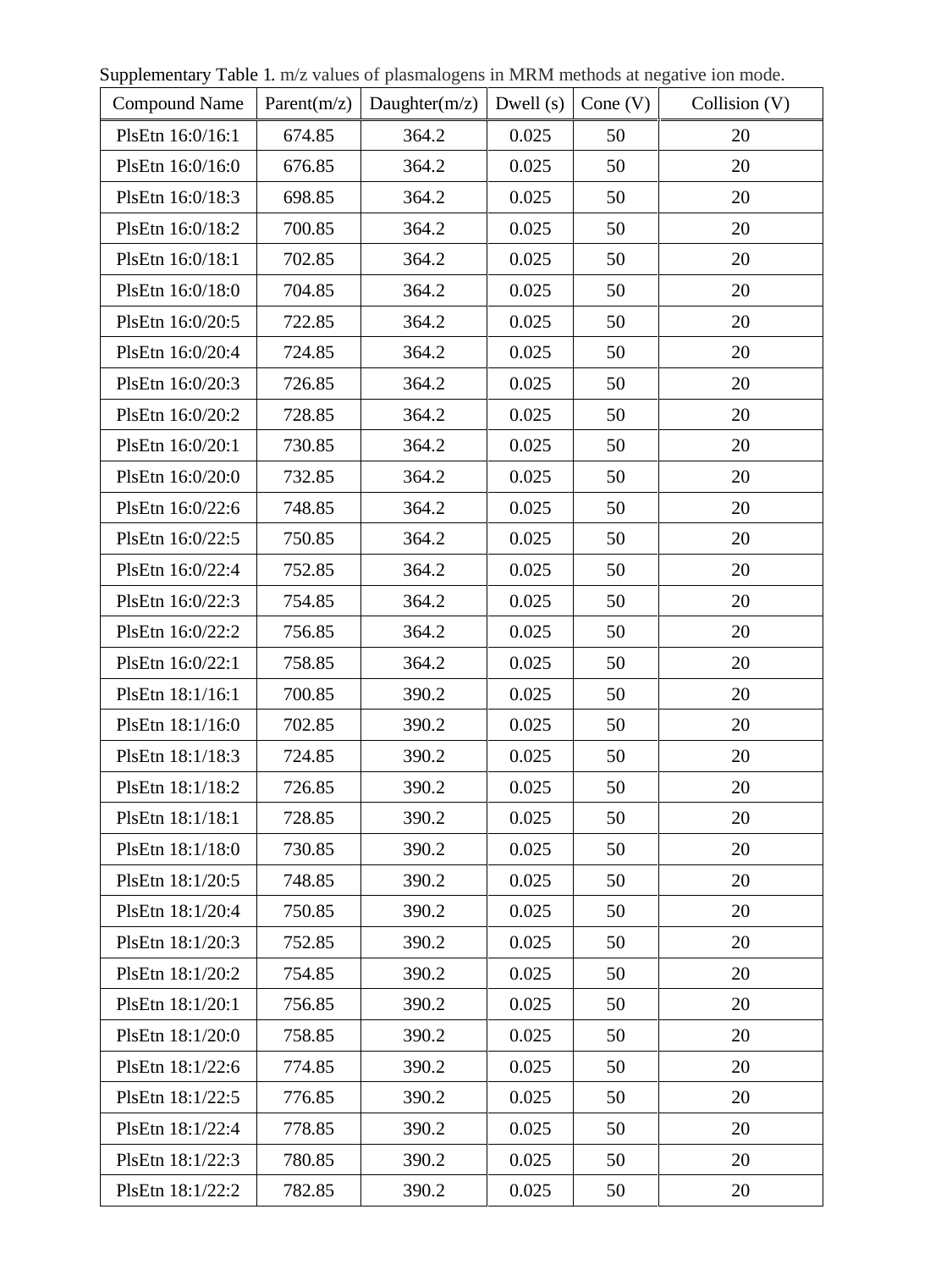

## Slide 4
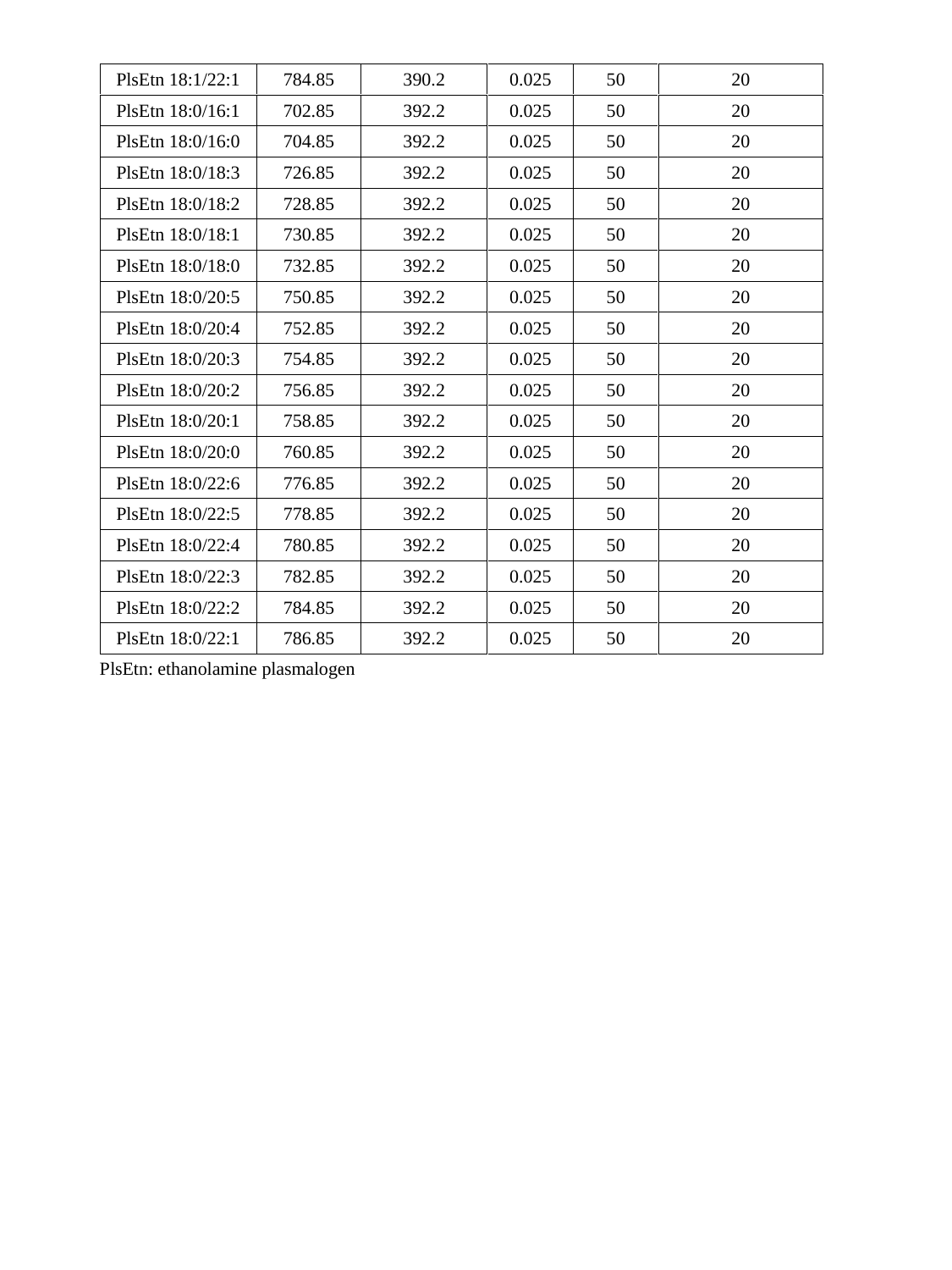

Supplement: Supplementary file 1 [file Presentation1.PPTX]
